# 

**Published:** 1883-09

**Authors:** 


					﻿SEPTEMBER, 1883.)
THIRTIETH YEAR.
IIATJJS
■ JOlJBNAIi 1B
OF
Oxjajj X XX
Guaranteed Circulation 200,000 Copies in 12‘ Months.
E. H. GIBBS, A.M., M.D.^ Editor,
No. 21 CLINTON PLACE, EIGHTH STREET NEW YORK.
TERMS: $1 a Year.
Single number, 10 cts.
				

